# EpCAM (CD326) finding its role in cancer

**DOI:** 10.1038/sj.bjc.6603758

**Published:** 2007-05-01

**Authors:** P A Baeuerle, O Gires

**Correction to**: *British Journal of Cancer* (2007) **96**, 417–423. doi: 10.1038/6603494

The authors of the above would like to rightfully credit the work of a researcher that they now feel should have been cited in their above paper in *BJC*. Figure 1 shows that EpCAM directly interacts with claudin-7; the direct interaction of EpCAM and claudin-7 had been previously unraveled by the researcher Markus Ladwein and his co-workers.

The full work was published in *Experimental Cell Research* in October 2005 (full reference shown below):

Ladwein M, Pape UF, Schmidt DS, Schnolzer M, Fiedler S, Langbein L, Franke WW, Moldenhauer G, Zoller M (2005) The cell–cell adhesion molecule EpCAM interacts directly with the tight junction protein claudin-7. *Exp Cell Res* 309(2): 345–357.

